# Optimizing Lung Collapse During One-Lung Ventilation: Physiological Mechanisms and Clinical Strategies: A Narrative Review

**DOI:** 10.3390/jcm15135078

**Published:** 2026-06-29

**Authors:** Sung-Hye Byun

**Affiliations:** Department of Anesthesiology and Pain Medicine, School of Medicine, Kyungpook National University, Kyungpook National University Chilgok Hospital, Daegu 41404, Republic of Korea; shbyun82@knu.ac.kr

**Keywords:** one-lung ventilation, lung isolation, lung collapse, thoracic anesthesia, double-lumen tube, bronchial blocker

## Abstract

Effective thoracic surgery requires timely, predictable operative lung collapse. During one-lung ventilation (OLV), lung collapse is not merely a mechanical consequence of nonventilated lumen opening but a phase-dependent physiological process. Rapid phase I collapse is driven by elastic recoil and passive gas venting, whereas slower phase II collapse depends on residual alveolar gas absorption. Communication between the operative-side airway and the atmosphere before pleural opening may permit tidal gas movement, ambient air entrainment, and nitrogen re-entry during the closed-chest period, delaying subsequent absorption collapse. This narrative review reorganizes lung collapse strategies, including denitrogenation, operative-side airway occlusion, preemptive OLV, disconnection, bronchial suction, and the open-clamp airway technique, according to timing and physiological target. Before pleural opening, alveolar nitrogen should be reduced and ambient air entrainment prevented. Around the pleural opening, airway patency and brief suspension of positive-pressure ventilation may preserve elastic recoil venting. During OLV maintenance, re-clamping or limiting atmospheric communication may support residual gas absorption. This phase-based framework interprets recent clinical findings as interventions acting before, during, and after pleural opening. This may help clinicians select strategies according to the lung isolation device, oxygenation reserve, and surgical environment, although standardized endpoints and component-level validation remain necessary.

## 1. Introduction

Lung isolation and one-lung ventilation (OLV) are fundamental techniques in thoracic anesthesia. They are widely used in pulmonary surgery, as well as in operations involving intrathoracic structures such as the esophagus, aorta, and thoracic spine, in which they facilitate surgical access and prevent contamination [1]. With the expansion of video-assisted thoracoscopic surgery (VATS) and robotic thoracic surgery, rapid and predictable collapse of the operative lung has become increasingly important for securing a restricted intrathoracic working space and maintaining procedural stability.

Physiological studies over the past two decades have shown that nonventilated lung collapse is not a simple mechanical event that follows the opening of the nonventilated lumen. Instead, it is a sequential process driven by distinct mechanisms over time [2]. Immediately after pleural opening, a relatively rapid phase I collapse occurs due to pulmonary elastic recoil, followed by a slower phase II collapse that is driven by residual gas absorption within the lung [3,4,5,6]. Accordingly, an effective lung collapse strategy requires an understanding of when the airway should be opened or closed and which physiological process should be preserved or facilitated at each time point.

From this perspective, the traditional practice of leaving the nonventilated lumen of a double-lumen tube (DLT) open to the atmosphere during the closed-chest period has been re-evaluated. During OLV, positive-pressure ventilation of the dependent lung may generate tidal gas movement (TGM) and ambient air entrainment in the nonventilated lung. In particular, re-entry of nitrogen may delay the absorptive removal of residual gas, consequently slowing lung collapse [4,7,8,9]. Therefore, several strategies have been recently studied, including airway occlusion [8,9], preemptive OLV [10], disconnection [11,12,13], suction [14], and denitrogenation [3,15]. Recent reviews have mainly organized these approaches by technique [16,17].

The open-clamp airway technique (OCAT) reported by Huang et al. [18] and the direct comparison of the disconnection technique versus preemptive OLV reported by Zhang et al. [19] indicate that the field is moving beyond a simple list of techniques toward a comparison of how each strategy acts at different phases of lung collapse. Although these studies primarily targeted different physiological mechanisms (support of phase I collapse versus preservation of phase II collapse), their timing of application spans the period before and after pleural opening and may influence both passive elastic recoil during phase I and residual gas absorption or diffusion related to phase II.

Therefore, this narrative review aims to reorganize the strategies for nonventilated lung collapse from a phase-specific perspective. While previous reviews have classified lung collapse-promoting techniques by maneuver, this review uses the timing of action and physiological factors that promote or interfere with collapse at each time point as the main decision axis. This approach may help integrate the apparent conflicting findings regarding disconnection duration and clarify the temporal scope and clinical meaning of phase II protection raised by recent protocols such as OCAT.

## 2. Two-Phase Physiology of Lung Collapse During OLV

When OLV is initiated, and the nonventilated airway communicates with the atmosphere, the nonventilated lung begins to deflate before the pleural cavity is opened. However, in a closed chest, this deflation occurs only to the extent permitted by persistent lung-chest wall mechanical coupling, and may bring the lung toward functional residual capacity (FRC). Therefore, it should be distinguished from the full operative lung collapse observed after pleural opening.

When the pleural cavity is opened, the transpulmonary distending pressure that maintains the inflation of the nonventilated lung is lost, and lung-chest wall coupling is released. The lung then undergoes an additional and immediate passive collapse owing to its intrinsic elastic recoil. This early phase I collapse generally progresses rapidly, within approximately 1 min, and represents the key time window during which the gas remaining in the nonventilated lung is passively vented through the airway device lumen [2,14].

After this rapid and early collapse, progressive small airway closure limits further passive gas venting through the airway. The gas remaining within the alveoli then diffuses into and is absorbed by the pulmonary blood flow, producing the slower phase II collapse [2,4]. This phase is influenced by the composition and solubility of the residual gas [3,5,15,20], maintenance of perfusion to the nonventilated lung, and additional inflow of ambient air. Nitrogen is substantially less soluble in the blood than oxygen or nitrous oxide and is therefore removed more slowly, which tends to maintain alveolar gas volume [20,21]. Moreover, as oxygen uptake proceeds and alveolar oxygen tension decreases, hypoxic pulmonary vasoconstriction may reduce blood flow to the nonventilated lung, potentially further slowing residual gas absorption. Therefore, nitrogen re-entry can delay phase II collapse through both gas solubility and perfusion-related mechanisms.

This two-phase physiology provides the basic framework for interpreting lung collapse strategies during OLV. Immediately after pleural opening, phase I depends on maximizing passive venting driven by elastic recoil; during phase II, the key objective is to minimize nitrogen-rich ambient air entrainment that interferes with residual gas absorption [3].

## 3. Why Can Opening the Nonventilated Lumen Before Pleural Opening Delay Collapse?

Opening the nonventilated lumen to the atmosphere may seem intuitively helpful because it provides a pathway for air to leave the lung. However, in the closed-chest period before the pleural cavity is opened, this intuition does not necessarily hold. Positive-pressure ventilation delivered to the dependent lung can be transmitted to the nonventilated lung through mediastinal displacement and pressure changes in the contralateral hemithorax. If the nonventilated lumen communicates with the atmosphere, the gas may move bidirectionally with each breath, leaving and then re-entering the nonventilated lung; this phenomenon is referred to as TGM [7]. Pfitzner et al. directly observed gas movement between the nonventilated lung and the external environment by connecting the nonventilated lung to a spirometer at OLV onset in patients undergoing VATS. This representative physiological study showed that nonventilated lumen opening did not simply provide a route for gas egress; in the closed chest, it also served as a pathway for the re-entry of ambient air, particularly nitrogen-containing air, into the nonventilated lung [7].

Wei et al. subsequently evaluated the closed-chest TGM concept proposed by Pfitzner as a patient-level physiological variable in a contemporary OLV cohort [22]. In 108 patients managed with a DLT, a pediatric spirometry sensor was connected to the nonventilated lumen. Immediately after OLV onset, the TGM volume of the nonventilated lung was 78 mL, and the TGM index, corrected for body surface area, was 45 mL/m^2^. The TGM index was negatively correlated with the oxygen concentration in the nonventilated lung 5 min after OLV. This supports the concept that passive ventilation caused by TGM can reintroduce nitrogen-containing ambient air and dilute the oxygen fraction in the nonventilated lung.

This phenomenon is not merely a device-specific issue. In a physiological comparison of DLTs and bronchial blockers (BBs), Moreault et al. reported similar gas movement patterns in both devices when the nonventilated lumen was connected to the atmosphere during OLV before pleural opening [8]. Airway pressure in the nonventilated lung became progressively negative in both the DLT and BB groups, consistent with ambient air entrainment toward the nonventilated lung. Thus, TGM and air entrainment before pleural opening should be understood as common physical phenomena that occur when a lung isolation device is connected to the atmosphere in the closed chest, rather than an issue limited to a specific device [8].

Furthermore, this phenomenon is important because it directly affects the physiology of phase II collapse. If the initial phase I collapse after pleural opening occurs rapidly through elastic recoil and passive venting, the subsequent phase II collapse depends on the absorption of residual alveolar gas. Oxygen can be absorbed into the blood relatively quickly, whereas nitrogen is poorly soluble and maintains alveolar gas volume for a longer period [21]. Repeated ambient air inflow into the nonventilated lung during the closed-chest period before pleural opening can therefore increase the nitrogen fraction in the nonventilated lung and delay subsequent absorption collapse.

These physiological observations have been translated into clinical strategies such as operative-side airway occlusion [9], preemptive OLV [10], and denitrogenation [3,5]. Accordingly, the key goal during the closed-chest period before pleural opening is not to open the nonventilated lumen to the atmosphere in advance, but to block ambient air and nitrogen re-entry and preserve conditions that are favorable for phase II absorption collapse.

## 4. Clinical Strategies Organized by Phase-Specific Timing

The main clinical strategies for improving nonventilated lung collapse can be divided into three groups according to the timing of their action (Table 1). The first group prepares and protects the conditions favorable for phase II absorption collapse during the closed-chest period. The second group maximizes passive venting driven by elastic recoil during the brief phase I window immediately after pleural opening. The third group extends the temporal scope of phase II protection to the post-opening OLV period. This section reorganizes the available evidence according to this timing-based classification and discusses a recent randomized controlled trial that directly compares two physiological targets (described in Section 4.4).

Each strategy should be assessed along two axes: the phase in which it acts and the strength of the supporting evidence. Some strategies map clearly to a collapse phase but have limited clinical evidence (e.g., bronchial suction), whereas others have a clear physiological target but have associated practical or clinical constraints that limit routine use (e.g., nitrous oxide). By explicitly separating these axes, this section aims to address not only what each technique involves but also where it acts and how it should be positioned in clinical practice.

### 4.1. Phase II Priming/Protection: Strategies During the Closed-Chest Period Before Pleural Opening

Strategies during the closed-chest period can be divided according to three physiological goals. First, OLV can be initiated before pleural opening to provide time for the absorption or diffusion of residual gas within the nonventilated lung. Second, the nonventilated lumen (the operative-side airway) can be isolated from the atmosphere to prevent ambient air entrainment and nitrogen re-entry. Third, the alveolar nitrogen concentration can be reduced before OLV onset, creating a gas composition favorable for subsequent absorption collapse.

Therefore, representative strategies in this phase include operative-side airway isolation, preemptive OLV, and denitrogenation. Nitrous oxide may be considered an adjunctive method that selectively accelerates absorption collapse during this period.

#### 4.1.1. Operative-Lumen Occlusion Before Pleural Opening: Airway Isolation Strategy

The purpose of operative-side airway isolation before pleural opening is not to actively collapse the nonventilated lung but to prevent communication between the nonventilated lung and the atmosphere during the closed-chest period. This blocks ambient air entrainment and nitrogen re-entry. Thus, this strategy does not contradict the principle that airway patency may be required for phase I venting immediately after pleural opening. Before pleural opening, airway isolation is important; immediately after pleural opening, adequate airway patency may be required to prevent impediment of the passive venting driven by elastic recoil.

Somma et al. clinically tested this physiological mechanism in a randomized controlled trial and reported that the lung deflation time was significantly shorter when the nonventilated DLT lumen was kept closed until pleural opening [9]. The median deflation time was 24 min in the occlusion group and 54 min in the control group. Gas exiting the nonventilated lung after pleural opening had a higher oxygen fraction in the lumen occlusion group, supporting the possibility that ambient air, particularly nitrogen-containing air, had entered the nonventilated lung in the control group [9].

The advantage of this strategy is that it does not require additional equipment or complex manipulation. Moreover, the finding by Moreault et al. regarding the similar gas movement patterns between DLTs and BBs suggests that blocking communication with the atmosphere before pleural opening is not a DLT-specific maneuver, but a general principle applicable across lung isolation devices [8].

#### 4.1.2. Preemptive OLV: Combining Early Timing with Airway Isolation

The main protocol feature of preemptive OLV is that it is initiated earlier than in the control condition. The goal is to provide additional time for phase II absorption or diffusion. However, preemptive OLV should not be understood simply as earlier initiation of OLV; it is more appropriately interpreted as a phase II priming strategy that combines early OLV timing with operative-side airway isolation. If the nonventilated lumen communicates with the atmosphere during the closed-chest period before pleural opening, initiating OLV earlier could simply prolong the duration of ambient air entrainment and nitrogen re-entry. Therefore, for preemptive OLV to be physiologically advantageous, early OLV should be combined with operative-side airway isolation in the clinical protocol.

A 2019 randomized controlled trial by Zhang et al. illustrated the clinical relevance of this combined strategy [10]. In the preemptive OLV group, OLV was initiated relatively early after lateral positioning and the operative side lumen remained closed until pleural opening. The time to satisfactory lung collapse was significantly shorter in the preemptive OLV group than in the control group (9.1 vs. 14.1 min), with no oxygenation deterioration observed in either group [10]. Although the control condition differed from that in the Somma et al. study, because OLV was initiated immediately before pleural opening, these findings support the concept that combining early OLV timing with airway isolation can facilitate lung collapse.

#### 4.1.3. Denitrogenation: Preparing the Gas Composition for Phase II Collapse

Reduction in the alveolar nitrogen fraction before the onset of OLV is another key component of phase II priming. Because phase II collapse progresses as residual alveolar gas is absorbed into the blood, the alveolar gas composition at the start of OLV can influence the subsequent rate of collapse. Compared with oxygen, nitrogen is less soluble in the blood and is absorbed more slowly; therefore, a higher alveolar nitrogen fraction maintains the residual gas volume for a longer period.

Ko et al. reported that the use of an inspired gas mixture containing air during the two-lung ventilation (TLV) phase delayed the subsequent lung collapse during OLV [3]. This study has been cited as a physiological basis for using a fraction of inspired oxygen (FiO_2_) of 1.0 during the TLV phase immediately before OLV to achieve denitrogenation. In numerous subsequent clinical studies evaluating lung collapse, application of an FiO_2_ of 1.0 during the TLV phase immediately before OLV can be interpreted as a protocol design based on the same phase II physiology [8,9,10,12,13,18,19,26]. Denitrogenation should therefore not be considered a specific adjunctive technique, but a basic phase II priming strategy for incorporation into most OLV protocols.

#### 4.1.4. Facilitating Phase II Absorption with Nitrous Oxide: Selective Application

Because nitrous oxide has greater blood solubility than oxygen or nitrogen [27], its presence in residual alveolar gas may accelerate absorption collapse. In a study using BBs, Yoshimura et al. reported that patients receiving 50% nitrous oxide during the TLV phase had higher lung collapse scores at 5 and 10 min after pleural opening than those receiving 100% oxygen [5]. This finding can be interpreted as facilitation of residual gas absorption due to the greater blood solubility of nitrous oxide; the mechanism may also be related to the second gas effect or concentration effect [23].

However, nitrous oxide has important limitations when used as a routine strategy. It must be considered in relation to the oxygenation reserve; concerns regarding alveolar stability, bowel gas expansion, or other contraindications [5,16]; and environmental burden [28]. Thus, nitrous oxide is best understood as a physiological tool that can selectively accelerate phase II absorption, whereas denitrogenation with an FiO_2_ of 1.0 during the TLV phase remains the more practical general approach.

### 4.2. Preservation and Adjunctive Support of Phase I Passive Venting: Strategies During Pleural Opening

The rapid phase I collapse observed in the operative field after pleural opening is caused by the intrinsic elastic recoil of the lung and generally occurs within approximately 1 min [2]. However, when interpreting the disconnection technique, phase I should not be considered as a post-pleural opening event only. Even in the closed chest, if positive-pressure ventilation is stopped and the nonventilated airway is opened, the nonventilated lung can first deflate toward the FRC within the limits imposed by the preserved lung-chest wall coupling. When the pleural cavity is opened, lung-chest wall coupling is released, additional recoil occurs, and rapid passive gas venting through the airway pathway completes the clinically observed phase I collapse [16].

Therefore, the time window addressed in this section should be considered not only as the first minute after pleural opening, but also as an elastic recoil window that spans the period immediately before and after pleural opening. This does not imply that the nonventilated lumen should remain open to the atmosphere throughout the closed-chest period; the basic principle during this period remains the prevention of ambient air entrainment and nitrogen re-entry. Disconnection should instead be interpreted as a brief, purpose-directed maneuver timed around pleural opening to reduce positive-pressure transmission and tidal volume trapping.

Clinical strategies in this phase have been developed in two directions: the disconnection technique, which preserves passive venting by reducing positive-pressure transmission, and bronchial suction, which actively augments venting by applying negative pressure.

#### 4.2.1. Disconnection Technique

The disconnection technique refers to the transient separation of the ventilator circuit or suspension of positive-pressure ventilation at the transition from TLV to OLV around the skin incision before or immediately after pleural opening. Its purpose is to allow the nonventilated lung to vent more effectively through elastic recoil. Its key concept is not active suction of the nonventilated lung, but reduction in positive-pressure transmission from the ventilated lung through the mediastinum to prevent impedance of phase I passive collapse [11,12,13,19].

Several randomized trials have shown that the disconnection technique can improve nonventilated lung collapse. Yoo et al. reported that combining disconnection with a BB produced better lung deflation than that with a BB alone [11]. Li et al. reported that a 2 min disconnection technique with a DLT accelerated the collapse of the nonventilated lung during thoracoscopic surgery [12]. Cheng et al. showed that combining a BB with a short disconnection technique achieved a collapse quality similar to that of a DLT, while preserving some of the advantages of the BB, such as smaller hemodynamic changes and a lower incidence of sore throat [13]. In a recent DLT-based study by Zhang et al., the disconnection technique also shortened the time to satisfactory collapse compared with conventional OLV [19].

The optimal duration of disconnection varies among studies, ranging from a brief disconnection to 2 min [11,12,13,19,29,30], and recent narrative reviews have identified the timing and duration of disconnection as unresolved issues [16]. These variations can be interpreted through the concept that elastic recoil operates across two stages around the pleural opening. If disconnection is applied before pleural opening, the nonventilated lung can deflate toward FRC while the chest remains closed, and tidal volume trapping at the onset of OLV may be reduced. If disconnection is applied immediately after pleural opening, the rapid phase I recoil that follows the release of pleural constraint can proceed without positive-pressure transmission. Clinically, the key variable may therefore not be the absolute duration of disconnection but how well the maneuver is synchronized with the elastic recoil window around the pleural opening.

From this perspective, even a short disconnection of 15 s may be effective if it is accurately applied immediately after pleural opening [13]. Conversely, a 2 min disconnection starting from the skin incision may include both closed-chest recoil before pleural opening and additional recoil after pleural opening, but it may also prolong apnea outside the rapid venting phase and cause transient hypercapnia [12]. Therefore, disconnection should not be applied as routine prolonged apnea, but as a short, purpose-directed maneuver timed to the phase I recoil window around the pleural opening.

The purpose of the disconnection technique differs somewhat between DLTs and BBs. With a DLT, passive venting through the relatively large operative side lumen is already available; therefore, the main role of disconnection is to reduce positive-pressure transmission and tidal volume trapping around the pleural opening, thereby preserving phase I elastic recoil collapse. In contrast, with a BB, the narrow and long internal channel may be a bottleneck for phase I gas egress. A recent review suggested that combining BB cuff deflation with disconnection allows the residual gas to exit through the larger single-lumen endotracheal tube rather than through the narrow BB channel [16]. In this context, disconnection with a BB can be considered as both a method for reducing positive-pressure transmission and as a rational device-specific maneuver that compensates for the structural limitations of the BB.

In summary, the disconnection technique is a representative strategy that supports phase I collapse. It is less invasive than bronchial suction and can be applied using both DLTs and BBs. However, its optimal timing and duration have not yet been standardized; therefore, disconnection should be tailored to the brief elastic recoil window around pleural opening, while considering the risks of oxygenation impairment and CO_2_ accumulation.

#### 4.2.2. Bronchial Suction

Bronchial suction aims to facilitate early lung collapse by actively extracting residual gas from the nonventilated lung with negative pressure during the brief phase I window immediately after pleural opening. Its main theoretical target is the early period before the occurrence of small airway closure. In this respect, it differs mechanistically from disconnection, which restores passive venting by reducing positive-pressure transmission and gas trapping.

The clinical effect of bronchial suction should be interpreted according to the lung isolation device and surgical setting. In the BB setting, especially with an Arndt blocker, suction may compensate for slow gas egress through the narrow and long blocker lumen, thereby facilitating lung collapse. El-Tahan reported that continuous bronchial suction shortened the time to total lung collapse compared with the disconnection technique in patients undergoing VATS managed with an Arndt blocker [24]. Subsequent randomized controlled trials of uniport VATS with BBs have also suggested that low negative pressure or suction of approximately 60 s can improve early lung collapse scores [25].

The evidence is limited in the DLT setting. Quan et al. randomized 80 patients undergoing VATS managed with DLTs to either 1 min of −30 cm H_2_O suction through the nonventilated lumen immediately after pleural opening or spontaneous collapse [14]. The suction group showed higher lung collapse scores at 1, 5, and 10 min; however, the difference at the primary endpoint of 10 min was only 0.5 points on a 10-point scale and did not reach the prespecified clinically meaningful difference of 1.0 points. Thus, in DLT-based OLV, bronchial suction may yield statistical improvement but has not shown sufficiently consistent and clinically meaningful benefit to justify its routine use.

From a safety perspective, the risk-benefit balance of suction depends on its timing, pressure, and duration. Suction before pleural opening has been associated with the possibility of increased negative pressure in the nonventilated lung, severe hypoxemia, and pulmonary edema [31]. Excessive negative pressure or prolonged suction also raises potential concerns regarding airway mucosal injury [32]. Additionally, a suction catheter may interfere with surgical manipulation or become involved in the bronchial staple line [33]. If suction is used, it should preferably be applied briefly immediately after pleural opening, followed by catheter removal.

Therefore, bronchial suction can be classified as a phase I strategy. However, in DLT-based OLV, it is better positioned as a selective adjunct for situations in which early surgical exposure is particularly important or spontaneous collapse is insufficient, rather than as a routine technique that replaces basic deflation strategies such as disconnection or occlusion/preemptive OLV. However, in BB or uniport VATS settings, evidence is accumulating, and recommendations should be interpreted separately according to the device and surgical approach.

### 4.3. Temporal Extension of Phase II Protection: OCAT

The closed-chest phase II preparation and protection strategies discussed above, including simple nonventilated lumen occlusion in the Somma et al. study and preemptive OLV with nonventilated lumen occlusion in the Zhang et al. study, share a common feature: communication with the atmosphere is blocked until pleural opening, followed by clamp release [9,10]. These approaches treat phase II protection as an issue that is limited to the closed-chest period. However, if the nonventilated lumen remains connected to the atmosphere after pleural opening, ambient air, particularly nitrogen-containing air, may repeatedly re-enter the nonventilated lung while residual gas absorption continues. From this perspective, OCAT, proposed by Huang et al., can be interpreted as a protocol that extends the temporal scope of phase II protection from the closed-chest period to the OLV maintenance period [18].

OCAT comprises two steps. First, immediately after pleural opening, both DLT limbs are opened to the atmosphere, and TLV is suspended for 1 min to facilitate phase I venting. Second, at the end of this 1 min interval, the nonventilated lumen is re-clamped, and OLV is initiated, with the clamp maintained during OLV. This second step extends the temporal application of the previous lumen occlusion strategy beyond pleural opening by limiting ambient air entrainment and nitrogen re-entry during phase II absorption collapse.

Pfitzner summarized the physiological operation of OCAT in three elements [6]: the nonventilated lung is (i) free from slowly diffusing nitrogen, (ii) allowed to collapse through elastic recoil via an open airway for 1 min after pleural opening, and (iii) progressively reduced in volume by continued oxygen uptake while the airway is occluded. The third element in particular shows that OCAT is not limited to phase I venting but that it also preserves phase II absorption collapse during OLV maintenance.

In a randomized trial of 60 patients, the OCAT group had a median time to complete lung collapse of 10 min, which was substantially shorter than the 25 min in the control group [18]. The incidence of complete collapse at 30 min after pleural incision and surgeon satisfaction were also higher, without an increase in intraoperative hypoxemia. These findings suggest that OCAT can induce rapid and stable lung collapse by sequentially combining phase I venting with phase II protection.

However, these OCAT findings should be interpreted with caution. The current evidence is based on a single-center, small-sample randomized trial. Although the integrated effect of OCAT has been demonstrated, the independent contributions of each component have not been isolated. It remains unclear whether the principal effect is derived from the 1 min open-airway suspension immediately after pleural opening, the nonventilated lumen occlusion maintained during OLV, or the sequential combination of both. Moreover, because the current evidence was generated mainly from DLT-based VATS, its generalizability to BB use, severe pleural adhesions, low oxygen reserves, and very short procedures remains uncertain. At the current level of evidence, OCAT is an integrated protocol requiring external validation; however, conceptually, it is important because it extends phase II protection from the closed-chest period to the OLV maintenance period.

### 4.4. Direct Comparison of Phase I Support and Closed-Chest Phase II Protection: Zhang et al. [19]

To date, most randomized trials have compared single strategies with conventional management. Consequently, the relative significance of phase I venting support, such as disconnection, and closed-chest phase II preservation, such as preemptive OLV or lumen occlusion, largely depends on indirect comparisons. In this regard, the 2025 randomized controlled trial by Zhang et al. is important because it directly compared phase I support with closed-chest phase II protection [19].

The study enrolled 97 patients and compared three groups: (1) a disconnection group (group D), in which a 2 min disconnection was performed from skin incision as a phase I support strategy; (2) a preemptive OLV group (group P), in which OLV was initiated immediately after lateral positioning and the operative side lumen was clamped until pleural opening, representing limited closed-chest phase II protection; and (3) a conventional group (group C), in which OLV was initiated at skin incision. The findings led to two important conclusions. First, the final time to satisfactory collapse was identical in groups D and P (15.7 min) and shorter than that in group C (21.7 min). This suggests that phase I venting support and closed-chest phase II priming/protection can achieve similar clinical effects for the endpoint of satisfactory collapse despite acting at different time points. Second, the collapse score at 1 min after pleural opening was higher in group P than in group D. The authors interpreted this as reflecting the formation of conditions favorable for residual gas absorption or diffusion during the closed-chest period in group P. This suggests that phase II protection can affect not only late collapse but also the early surgical field immediately after pleural opening. Therefore, phase II priming can contribute to early collapse by lowering lung volume before the point at which phase I collapse is observed.

This study indicates that phases I and II are not completely independent clinical time segments but continuous processes that modify the starting conditions of the next phase. Disconnection and preemptive OLV with occlusion have different primary physiological targets; however, both influence the quality of lung collapse observed after pleural opening. Thus, the question is not only which phase a strategy targets but also when that strategy begins and how it affects the conditions of the next phase.

The relationship with OCAT raises additional research questions. Compared with OCAT, group P in the Zhang et al. study showed significant differences. In group P, the operative side lumen was occluded only during the closed-chest period before pleural opening, and the clamp was released at pleural opening. Thus, the phase II protection in group P was limited to the closed-chest period and was not an extended phase II protection strategy in which nonventilated lumen occlusion was maintained during OLV, as in OCAT. Additionally, group P did not include an active phase I venting step, such as the 1 min bilateral open-airway suspension immediately after pleural opening used in OCAT. These differences lead to the next research focus: the clinical effect of OCAT should be dissected to determine which component is derived from phase I venting immediately after pleural opening and which is derived from sustained phase II protection during OLV.

## 5. Phase-Based Clinical Practice Algorithm

When applying the available evidence to clinical practice, the key is not to select one technique uniformly but to identify the current physiological target relative to pleural opening. The following clinical practice algorithm is proposed:

**During the TLV phase before OLV:** When feasible, denitrogenation with an FiO_2_ of 1.0 should be used to reduce the nitrogen burden of the residual gas. Nitrous oxide may facilitate absorption collapse, but it should be interpreted as a selective adjunct rather than a routine strategy.

**After lung isolation but before pleural opening:** The nonventilated lumen should be isolated from the atmosphere. The goal in this period is not active lung collapse, but the prevention of ambient air entrainment and nitrogen re-entry in order to maintain conditions favorable for absorption collapse.

**Around the pleural opening:** Passive venting should not be impeded during the brief elastic recoil window. Disconnection can be used as a maneuver timed to this window to reduce positive-pressure transmission and tidal volume trapping. Suction may be considered selectively, depending on the device and surgical situation.

**After pleural opening/OLV maintenance:** Strategies that combine an open-airway phase immediately after pleural opening with subsequent re-clamping, such as OCAT, are promising. However, at this stage, they should be viewed as integrated protocols requiring component-level validation rather than as universal standards.

The elements of this algorithm differ in their ease of implementation in routine clinical practice. Denitrogenation with an FiO_2_ of 1.0 before OLV and occlusion of the operative-side lumen until pleural opening can be incorporated into most clinical workflows without major changes. In contrast, timing the disconnection to the pleural opening and the 1 min open-airway period followed by re-clamping in OCAT requires close coordination with the surgical team. These maneuvers may therefore be more difficult to standardize in centers without dedicated thoracic anesthesia teams. A predefined verbal cue at the pleural opening or a brief checklist may help ensure consistent timing.

The practical application of this algorithm should be adjusted according to chronic obstructive pulmonary disease, pleural adhesion, low oxygen reserve, BB use, and specific surgical settings such as uniportal VATS, in which early exposure is particularly important.

## 6. Limitations and Future Directions

The current literature provides important evidence for interpreting nonventilated lung collapse strategies during OLV in a phase-specific manner. However, several limitations must be addressed before these strategies can be generalized as standardized clinical algorithms.

First, the endpoints for assessing lung collapse were inconsistent among the studies. They have included time to satisfactory collapse [10,19], time to complete collapse [18,24,26], lung collapse scores at selected time points [10,13,14,18,19,26], surgeon-rated exposure or satisfaction [12,18,19,24], hypoxemia [10,12,13,14,18,19,24], hypercapnia [12,19], and rescue maneuvers [12,13,24]. However, these outcomes differ among studies regarding their definition, timing of assessment, and rating scale, making direct comparison difficult. Therefore, future studies should report not only collapse scores and time to collapse but also surgeon-rated exposure, the need for rescue maneuvers such as continuous positive airway pressure or intermittent TLV, and safety outcomes, including hypoxemia and hypercapnia, using prespecified and standardized definitions and time points.

Second, the effect of each strategy may vary according to the device, patient characteristics, and surgical environment. Comparative studies on DLTs and BBs [8,26] and device-specific strategies [11,13] have also been reported. However, maneuvers such as disconnection, suction, and airway occlusion may have different implications depending on the lumen diameter [12,13], suction route [14,24,25], and a gas egress pathway. Furthermore, most studies have been single-center, small-sample trials conducted mainly in patients with relatively preserved pulmonary function undergoing VATS [10,12,13,14,18,19,25]. Therefore, caution is required when extrapolating these findings to patients with severe chronic obstructive pulmonary disease, obesity, pleural adhesion, or low oxygen reserve, as well as to surgical environments in which early exposure is important, such as uniportal VATS [25,34], or to robotic thoracic surgery, where CO_2_ insufflation and prolonged OLV may be relevant [35]. Future studies should investigate how the same phase-based strategy should be adapted across devices and patient phenotypes.

Third, in integrated protocols such as OCAT, the independent contribution of each component remains unclear [6,18]. OCAT combines phase I venting through open-airway suspension immediately after pleural opening with subsequent limitation of ambient air re-entry by re-clamping the nonventilated lumen during OLV [18]. However, current evidence does not determine whether the early open-airway interval, subsequent re-clamping, or the sequential combination of both components contributes most to the clinical effect [6,18,19]. Moreover, after small airway closure, air re-entry into already closed lung units is limited; therefore, the effect of post-opening re-clamping is better interpreted as limiting air re-entry into residual communicating or intermittently reopening units, rather than as acting on all already closed lung units [2,20,21]. Future studies should separately evaluate closed-chest occlusion, pleural opening open-airway suspension or disconnection, and post-opening re-clamping.

In summary, the clinical effects of several lung collapse strategies have been reported in the literature. However, heterogeneity in endpoints, devices, timing of application, and patient populations limits the integration of this data into a single phase-based clinical approach. Future studies should be designed not only to introduce additional maneuvers, but also to determine which components of existing strategies produce clinical benefits and under which clinical conditions these effects are reproducible.

## 7. Conclusions

Collapse of the nonventilated lung during OLV is not a simple mechanical phenomenon that progresses when the nonventilated airway is left open. Rather, it is a physiological process in which pressure conditions, airway communication, residual gas composition, and gas absorption interact over time, before and after pleural opening. Therefore, lung collapse strategies should be understood along a temporal axis as follows: before pleural opening, ambient air entrainment and nitrogen re-entry should be limited; around pleural opening, passive venting driven by elastic recoil should be preserved; and thereafter, conditions that allow residual gas absorption to translate into actual volume reduction should be maintained.

This phase-based framework repositions denitrogenation, airway occlusion, preemptive OLV, disconnection, suction, and OCAT, not as mutually competing techniques, but as strategies with different physiological targets acting in distinct time windows. However, integrated protocols such as OCAT have not yet isolated the individual contributions of the open-airway phase and the subsequent re-clamping phase. Future studies should therefore validate the timing of each strategy, device-specific interpretation, and reproducibility across patient phenotypes in a standardized manner.

## Figures and Tables

**Table 1:** Main Clinical Strategies for Optimizing Lung Collapse During OLV According to Phase-Specific Timing.

Strategy	Main Phase of Action	Timing/Airway State	Key Physiological Target	Representative Evidence	Practical Interpretation
Denitrogenation (TLV FiO_2_ 1.0)	Phase II priming	TLV phase before OLV	Reduces the nitrogen content/fraction of residual gas in the nonventilated lung, creating a gas composition favorable to subsequent absorption collapse.	Ko et al. 2009 [3]; Pfitzner et al. 2001 [2]	Basic preparatory strategy. Applicable regardless of the lung isolation device.
Nitrous oxide inhalation (e.g., 50%)	Phase II absorption facilitation	Selective closed-chest/pre-OLV interval	Facilitates residual gas absorption by exploiting the high blood solubility of nitrous oxide and the second gas effect.	Yoshimura et al. 2014 [5]; Liang et al. 2020 [23]; Pfitzner et al. 2001 [15]	Physiologically plausible but not routinely recommended because of oxygenation, contraindication, and environmental concerns.
Operative-side airway occlusion before pleural opening	Closed-chest phase II protection	From confirmation of lung isolation until pleural opening; nonventilated lumen closed	Blocks ambient air entrainment and nitrogen re-entry in the closed chest, preserving conditions favorable to residual gas absorption.	Somma et al. 2021 [9]; Moreault et al. 2021 [8]	The simplest baseline strategy: keep the operative-side airway closed before pleural opening, regardless of OLV timing.
Preemptive OLV with operative-side airway occlusion	Closed-chest phase II priming + protection	OLV started after lateral positioning or before pleural opening; operative-side lumen closed until pleural opening	Provides time for residual gas absorption/diffusion during the closed-chest period while blocking atmospheric re-entry.	Zhang et al. 2019 [10]; Somma et al. 2021 [9]; Moreault et al. 2021 [8]; Zhang et al. 2025 [19]	Meaningful only when early OLV is combined with airway isolation, rather than considered simply as earlier initiation of OLV.
Disconnection technique	Phase I passive venting preservation	Elastic-recoil window around pleural opening; transient cessation of positive-pressure ventilation or circuit disconnection	Reduces tidal volume trapping and positive-pressure transmission, supporting closed-chest recoil toward FRC and rapid passive venting after pleural opening.	Yoo et al. 2014 [11]; Li et al. 2017 [12]; Cheng et al. 2020 [13]; Zhang et al. 2025 [19]	Synchrony with the recoil window may be more important than absolute duration. Prolonged apnea and hypercapnia should be considered.
Bronchial suction	Phase I venting adjunct	Briefly immediately after pleural opening, before small airway closure	Uses negative pressure for active gas extraction and supports early gas egress when passive venting is insufficient.	El-Tahan 2015 [24]; Quan et al. 2017 [14]; Li et al. 2024 [25]	Evidence for routine use with DLTs is limited. May be considered as a selective adjunct with BBs or in uniport VATS. Pressure, duration, and safety remain issues.
OCAT (open-clamp airway technique)	Phase I venting preservation + extended phase II protection	Both DLT limbs open with TLV suspension for 1 min immediately after pleural opening, followed by re-clamping of the nonventilated lumen during OLV	Allows early passive venting driven by elastic recoil, then blocks ambient air/nitrogen re-entry during OLV maintenance to preserve absorption collapse.	Huang et al. 2024 [18]; Pfitzner 2024 commentary [6]	Promising integrated protocol. Based on a single RCT; the separate contributions of the phase I open-airway component and prolonged occlusion component require validation.

BB, bronchial blocker; DLT, double lumen tube; FiO_2_, fraction of inspired oxygen; FRC, functional residual capacity; OLV, one-lung ventilation; TLV, two-lung ventilation; RCT, randomized controlled trial; VATS, video-assisted thoracoscopic surgery.

## Data Availability

No new data were created or analyzed in this study.

## Abbreviations

The following abbreviations are used in this manuscript:

BB	bronchial blocker
CO_2_	carbon dioxide
DLT	double-lumen tube
FiO_2_	fraction of inspired oxygen
FRC	functional residual capacity
OCAT	open-clamp airway technique
OLV	one-lung ventilation
RCT	randomized controlled trial
TGM	tidal gas movement
TLV	two-lung ventilation
VATS	video-assisted thoracoscopic surgery
